# Supplementary material for: Biosynthesis of plant hemostatic dencichine in Escherichia coli
Source: Nat Commun. 2022 Sep 19;13:5492. doi: 10.1038/s41467-022-33255-3 (PMC9485241; doi:10.1038/s41467-022-33255-3)
Supplement: Supplementary file 1 — Supplementary information [file 41467_2022_33255_MOESM1_ESM.pdf]

Biosynthesis of plant hemostatic dencichine in *Escherichia coli*

Li *et al.*

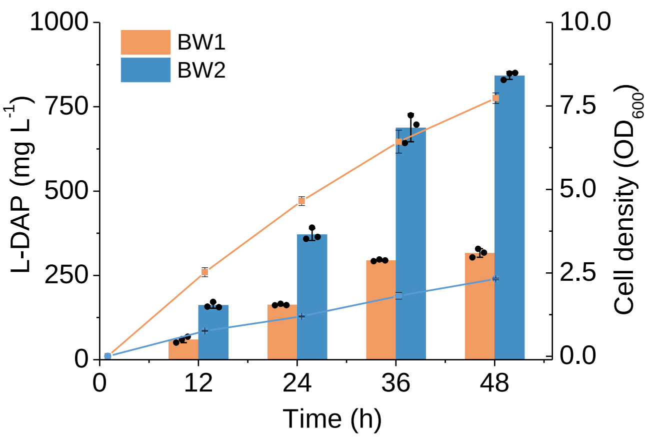

Supplementary Figure 1. Production and optimization of *L*-DAP by strain BW1 and BW2. The bars indicate the titer of *L*-DAP and the lines indicate biomass at OD_{600}. Data shown are mean ± SD (n=3 independent experiments). Source data are provided as a Source Data file.

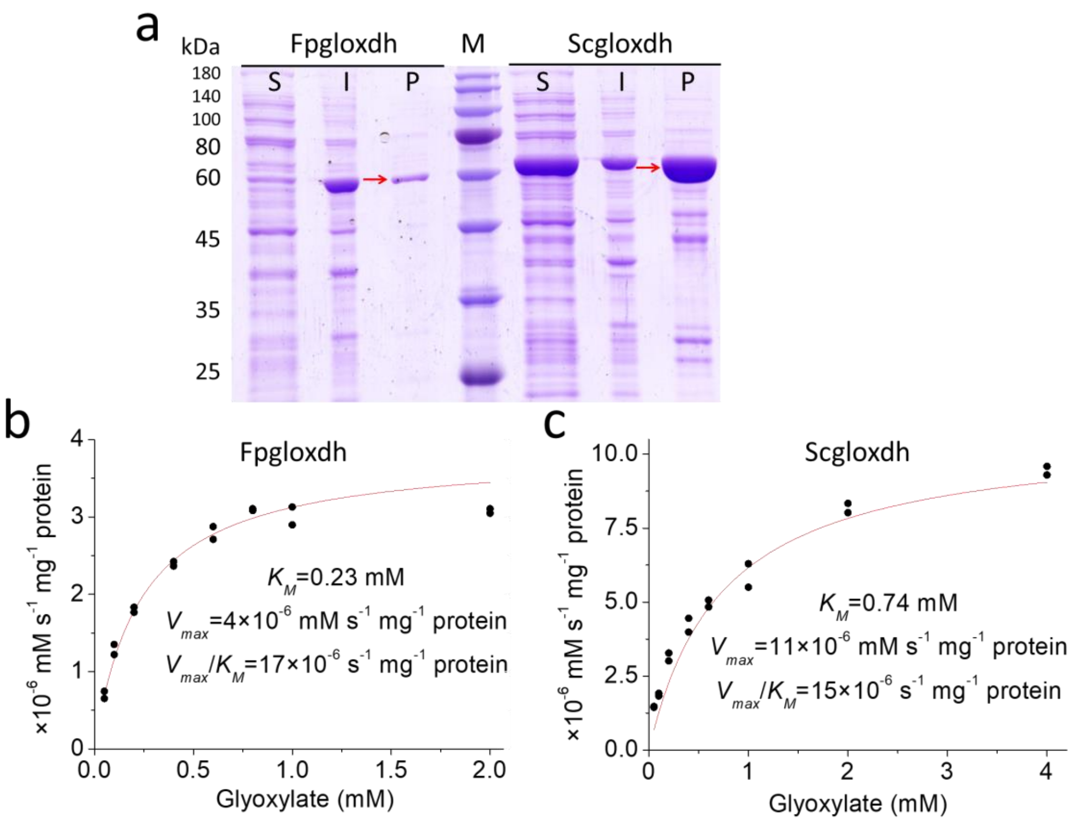

Supplementary Figure 2. Biochemical analysis of Fpgloxdh and Scgloxdh. a SDS-PAGE analysis of nickel-affinity purified codon-optimized His-Fpgloxdh and wild-type His-Scgloxdh. This experiment was repeated independently twice with similar results. **b, c** Kinetic analysis of Gloxdh over a gradient of glyoxylate concentrations (n=2, data distribution is less than 7.84% in duplicate experiments). Enzyme activity was determined by measurement the production of cytochrome *c* reduced at 550 nm. M, marker II; S, soluble fraction; I, insoluble fraction; P, purified protein. Source data are provided as a Source Data file.

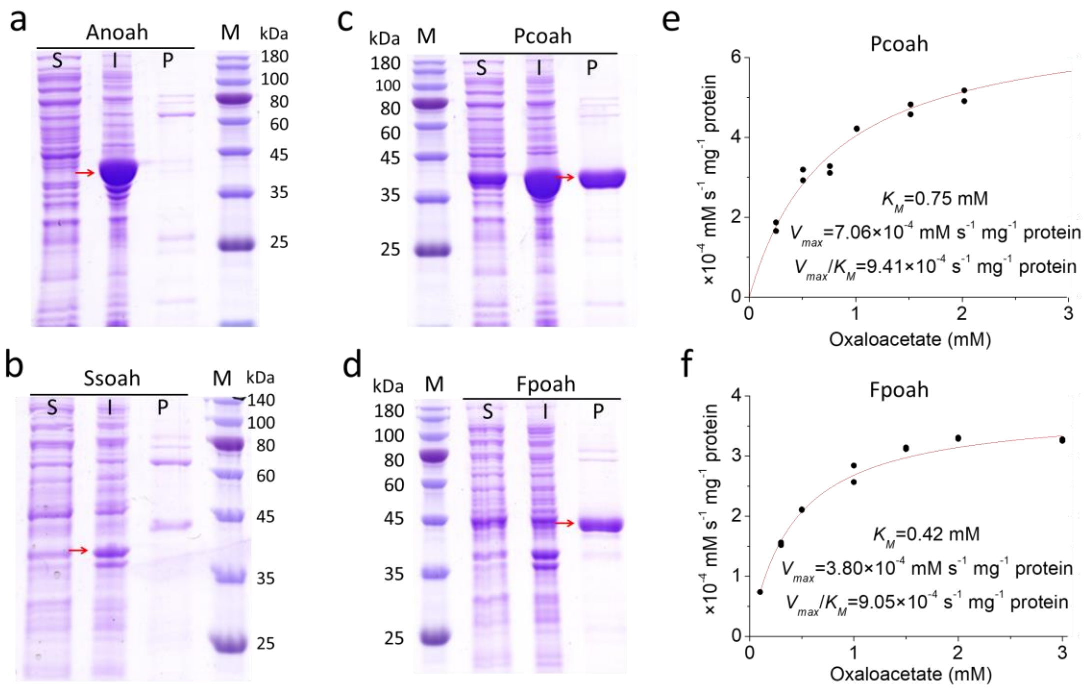

Supplementary Figure 3. Biochemical analysis of Anoah, Ssoah, Pcoah and Fpoah. a, b, c, d SDS-PAGE analysis of nickel-affinity purified Anoah, Ssoah, Pcoah and Fpoah. This experiment was repeated independently twice with similar results. **e, f** Kinetic analysis of Oah over a gradient of oxaloacetate concentrations (n=2, data distribution is less than 6.13% in duplicate experiments). Enzyme activity was determined by measurement the consumption of oxaloacetate at 255 nm. M, marker II; S, soluble fraction; I, insoluble fraction; P, purified protein. Source data are provided as a Source Data file.

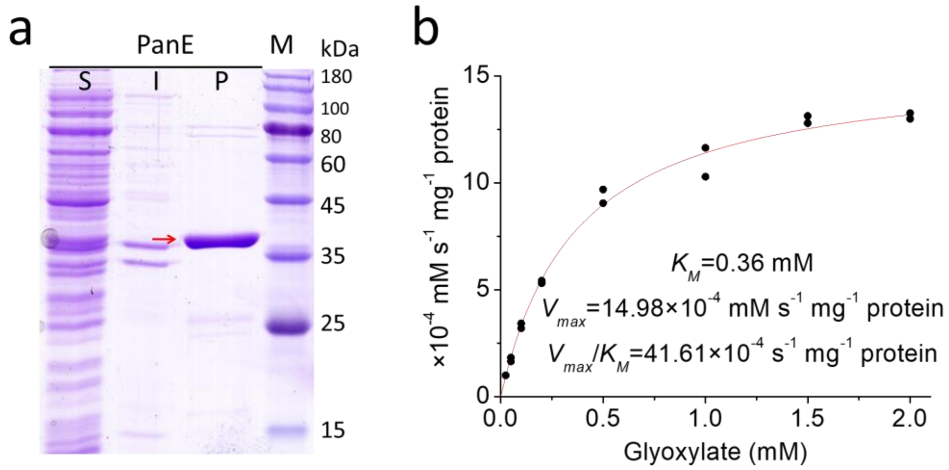

Supplementary Figure 4. Biochemical analysis of PanE. a SDS-PAGE analysis of nickel-affinity purified PanE. This experiment was repeated independently twice with similar results. **b** Kinetic analysis of PanE over a gradient of glyoxylate concentrations (n=2, data distribution is less than 6.16% in duplicate experiments). Enzyme activity was determined by measurement the production of NADPH at 340 nm. M, marker II; S, soluble fraction; I, insoluble fraction; P, purified protein. Source data are provided as a Source Data file.

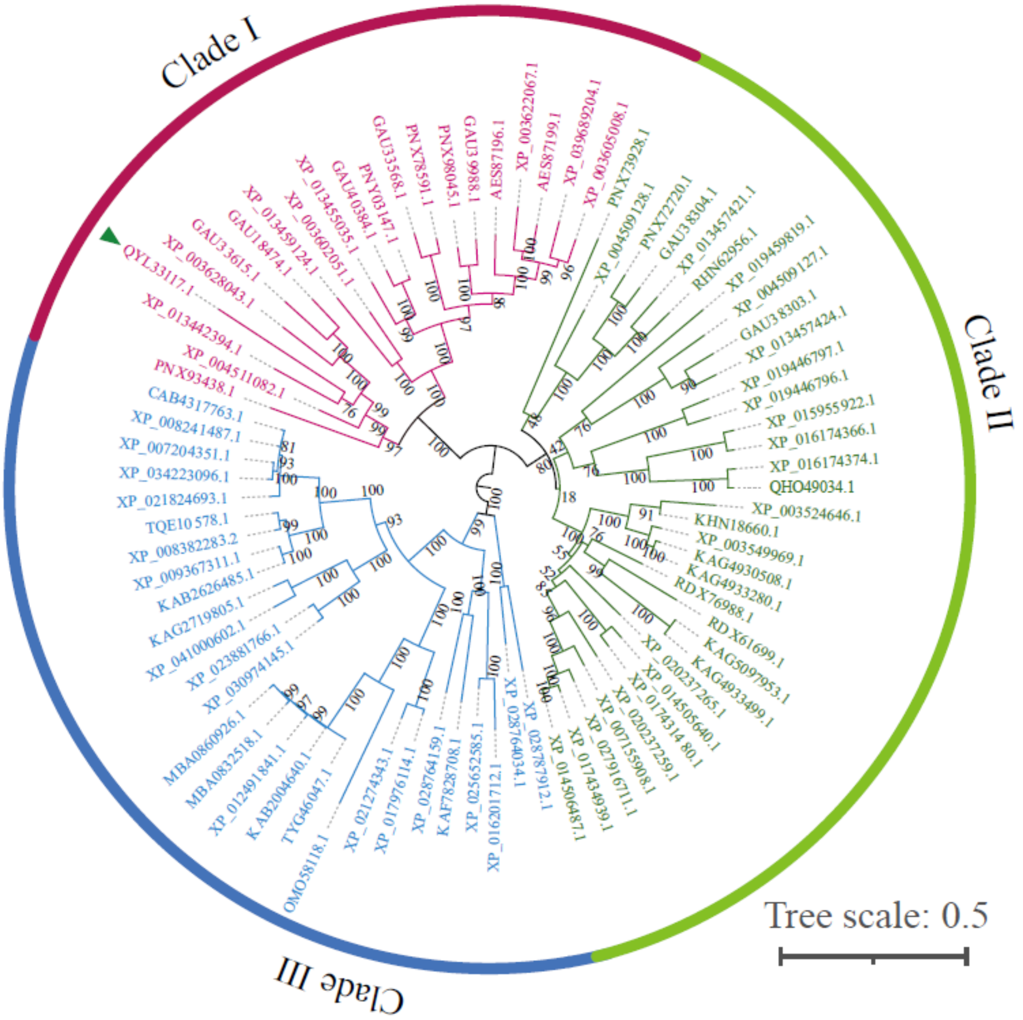

Supplementary Figure 5. Phylogenic relationships of LsBAHD in *L. sativus* with sequences encoding highly homologous proteins. Amino acid sequences were aligned and the Maximum Likelihood (ML) tree was built using MAFFT (version 7.407). The phylogeny was constructed with 1000 implementations of ultrafast bootstrap tests with IQ-TREE. The Model of substitution is JTT+I+G4 (Jones-Taylor-Thornton model, Has Invariant sites plus discrete Gamma model). The tree was further annotated by iTOL (Interactive Tree Of Life).

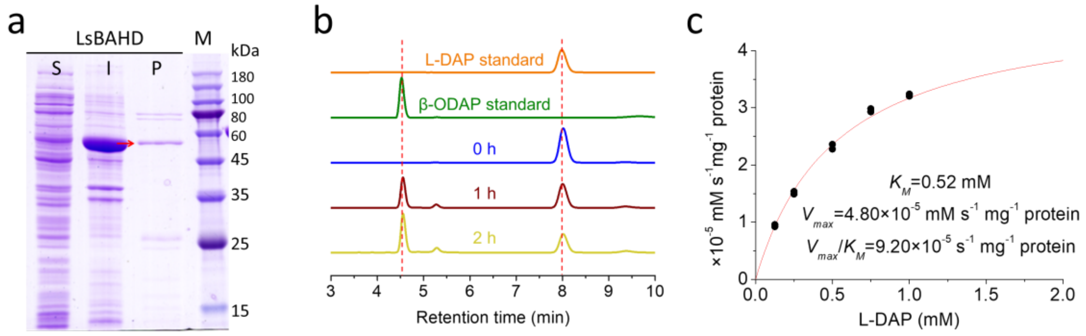

Supplementary Figure 6. Biochemical analysis of LsBAHD. a SDS-PAGE analysis of nickel-affinity purified LsBAHD. This experiment was repeated independently twice with similar results. **b** The *in vitro* assay substrates and products were monitored by HPLC analysis. A PanE-LsBAHD coupling assay was used due to the chemical oxalyl-CoA is not commercially available. From top to bottom are L-DAP standard, β-ODAP standard and the reaction at 0 h, 1 h and 2 h, respectively. **c** Kinetic analysis of LsBAHD over a gradient of L-DAP concentrations (n=2, data distribution is less than 1.97% in duplicate experiments). Enzyme activity was determined by measurement the production of NADPH at 340 nm. M, marker II; S, soluble fraction; I, insoluble fraction; P, purified protein. Source data are provided as a Source Data file.

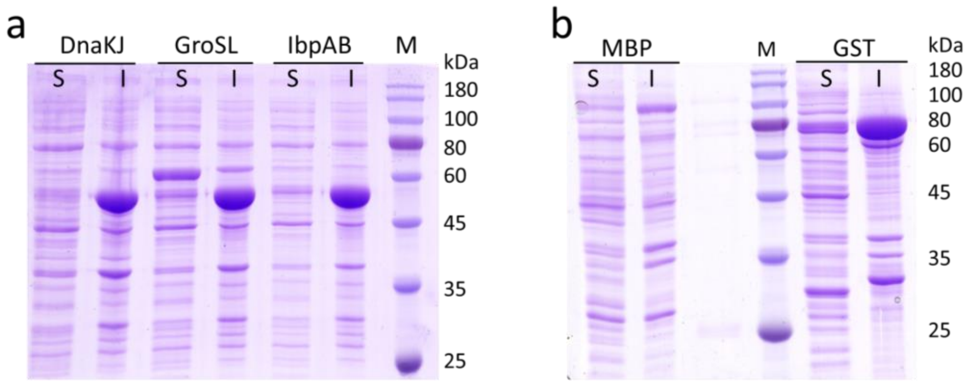

Supplementary Figure 7. Solubility optimization of LsBAHD by conventional methods. Expression levels of **a** LsBAHD co-expressed with DnaKJ, GroSL and IbpAB and **b** fusion enzymes (MBP: maltose-binding protein-LsBAHD and GST: glutathione S-transferase) were identified through SDS–PAGE analysis. M, marker II; S, soluble fraction; I, insoluble fraction. This experiment was repeated independently twice with similar results. Source data are provided as a Source Data file.

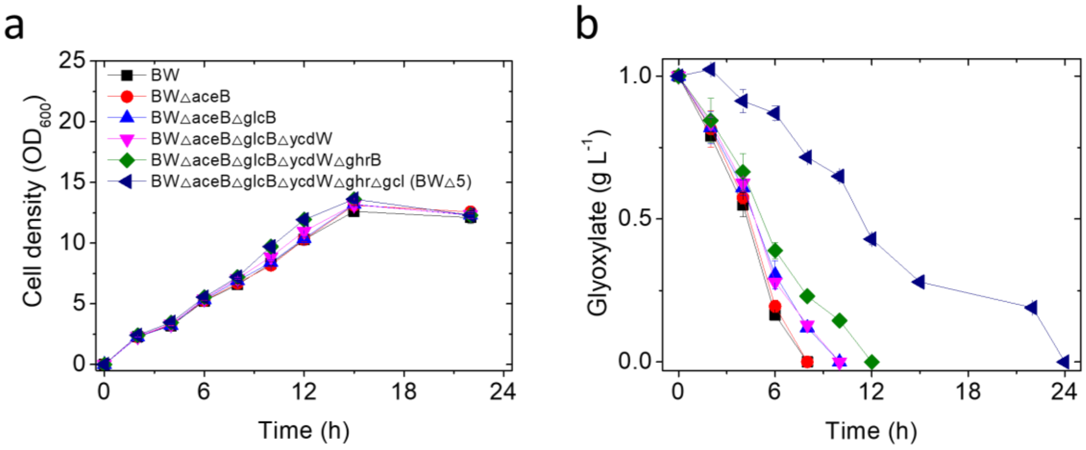

Supplementary Figure 8. Block the degradation pathways of glyoxylate. a Cell growth with time. **b** Glyoxylate consumption with time. Data shown are mean ± SD (n=3 independent experiments). Source data are provided as a Source Data file.

Supplementary Table 1. Strains used in this study.

Strains	Description	Source
E. coli BW25113	*rrnBT*14 Δ*lacZWJ*16 *hsdR*514 Δ*araBADAH*33 Δ*rhaBADLD*78	Coli genome stock center
E. coli XL-1 Blue	*recA1 endA1gyrA96thi-1hsdR17supE44relA1lac*	Stratagene
E. coli BL21 Star (*DE3*)	F^- *ompT* $hsdS_B$ $(r_B^- m_B^-)$ *gal dcm (DE3)*	Invitrogen
BW1	BW25113, pZE-sbnAB	This study
BW2	BW25113Δ*serB*, pZE-sbnAB	This study
BW3	BW25113, pCS-LsBAHD-panE	This study
BW4	BW25113, pCS-LsBAHD-ScAAE-Scgloxdh	This study
BW5	BW25113, pCS-LsBAHD-ScAAE-Fpoah	This study
BW6	BW25113, pCS-LsBAHD*-panE	This study
BW7	BW25113, pCS-LsBAHD*-ScAAE-Scgloxdh	This study
BW8	BW25113, pCS-LsBAHD*-ScAAE-Fpoah	This study
BWΔ5	BW25113Δ*aceB*Δ*glcB*Δ*ycdW*Δ*ghrB*Δ*gcl*	This study
BW9	BWΔ5, pCS-LsBAHD*-panE	This study
BW10	BWΔ5, pCS-LsBAHD*-ScAAE-Scgloxdh	This study
BW11	BWΔ5, pZE-sbnAB, pCS-LsBAHD*-panE	This study
BW12	BWΔ5, pZE-sbnAB, pCS-LsBAHD*-ScAAE-Scgloxdh	This study
BW13	BW25113, pZE-sbnAB, pCS-LsBAHD*-ScAAE-Fpoah	This study
BW14	BWΔ5, pZE-sbnAB, pCS-LsBAHD*-ScAAE-Fpoah-Scgloxdh	This study
BW15	BWΔ5Δ*serB*, pZE-sbnAB, pCS-LsBAHD*-ScAAE-Fpoah-Scgloxdh	This study
BW16	BWΔ5Δ*serB*, pZE-sbnAB, pCS-LsBAHD*-ScAAE-Fpoah-Scgloxdh, pSA-aceA	This study

Supplementary Table 2. Plasmids used in this study.

**Plasmids**	**Description***	**Source**
pZE12-luc	$P_L$lacO1, *colE ori*, *luc*, *Amp^R^*	Ref. 1
pCS27	$P_L$lacO1, *p15A ori*, *Kan^R^*	Ref. 1
pSA74	$P_L$lacO1, *pSC101 ori*, *Cl^R^*	Ref. 1
pETDuet-1	PT7, *pBR322 ori*, *Amp^R^*	Ref. 2
pMAL-c2x	Ptac, *pBR322 ori*, MBP tag, *Amp^R^*	Ref. 3
pGEX-6p-1	Ptac, *pBR322 ori*, GST tag, *Amp^R^*	Solarbio
pZE-sbnAB	pZE12-luc, *sbnA* and *sbnB* from *S. aureus*	This study
pET-Fpgloxdh	pETDuet-1, *Fpgloxdh* from *F. palustris*	This study
pET-Scgloxdh	pETDuet-1, *Scgloxdh* from *S. cerevisiae*	This study
pET-Anoah	pETDuet-1, *Anoah* from *A. niger*	This study
pET-Pcoah	pETDuet-1, *Pcoah* from *P. chrysogenum*	This study
pET-Fpoah	pETDuet-1, *Fpoah* from *F. palustris*	This study
pET-Ssoah	pETDuet-1, *Ssoah* from *S. sclerotiorum*	This study
pET-panE	pETDuet-1, *panE* from *M. extorquens*	This study
pET-LsBAHD	pETDuet-1, *Lsbahd* from *L. sativus*	This study
pCS-dnaKJ	pCS27, *dnaK* and *dnaJ* from *E. coli*	This study
pCS-groSL	pCS27, *groS* and *groL* from *E. coli*	This study
pCS-ibpAB	pCS27, *ibpA* and *ibpB* from *E. coli*	This study
pMAL-LsBAHD	pMAL-c2x, *Lsbahd*	This study
pGEX-LsBAHD	pGEX-6p-1, *Lsbahd*	This study
pET-LsBAHD*	pETDuet-1, *Lsbahd*	This study
pCS-LsBAHD-panE	pCS27, *Lsbahd* and *panE*	This study
pCS-LsBAHD-ScAAE-Scgloxdh	pCS27, *Lsbahd*, *ScAAE* and *Scgloxdh*	This study
pCS-LsBAHD-ScAAE-Fpoah	pCS27, *Lsbahd*, *ScAAE* and *Fpoah*	This study
pCS-LsBAHD*-panE	pCS27, *Lsbahd** and *panE*	This study
pCS-LsBAHD*-ScAAE-Scgloxdh	pCS27, *Lsbahd**, *ScAAE* and *Scgloxdh*	This study
pCS-LsBAHD*-ScAAE-Fpoah	pCS27, *Lsbahd**, *ScAAE* and *Fpoah*	This study
pCS-LsBAHD*-ScAAE-Fpoah-Scgloxdh	pCS27, *Lsbahd**, *ScAAE*, *Fpoah* and *Scgloxdh*	This study
pSA-aceA	pSA74, *aceA*	This study

*Amp^R, ampicillin resistant; Kan^R, kanamycin resistant; Cl^R, chloramphenicol resistant.

Supplementary references

1.Lin Y, Sun X, Yuan Q, Yan Y. Extending shikimate pathway for the production of muconic acid and its precursor salicylic acid in *Escherichia coli*. *Metab. Eng.* **23**, 62-69 (2014).

2.Shen X, *et al.* Elevating 4-hydroxycoumarin production through alleviating thioesterase-mediated salicoyl-CoA degradation. *Metab. Eng.* **42**, 59-65 (2017).

3.Chen Z, Li Y, Sun X, Yuan Q. Improvement of expression level of polysaccharide lyases with new tag GAPDH in *E. coli*. *J. Biotechnol.* **236**, 159-165 (2016).
