# Supplementary material for: Biosynthesis of plant hemostatic dencichine in Escherichia coli
Source: Nat Commun. 2022 Sep 19;13:5492. doi: 10.1038/s41467-022-33255-3 (PMC9485241; doi:10.1038/s41467-022-33255-3)
Supplement: Supplementary file 3 — Description of Additional Supplementary Files [file 41467_2022_33255_MOESM3_ESM.pdf]

File Name: Supplementary Data 1

Description: ESI-MS results of β-ODAP standard and sample.

File Name: Supplementary Data 2

Description: Oligonucleotides used in this study.
